# Design Two Novel Tetrahydroquinoline Derivatives against Anticancer Target LSD1 with 3D-QSAR Model and Molecular Simulation

**DOI:** 10.3390/molecules27238358

**Published:** 2022-11-30

**Authors:** Yongtao Xu, Baoyi Fan, Yunlong Gao, Yifan Chen, Di Han, Jiarui Lu, Taigang Liu, Qinghe Gao, John Zenghui Zhang, Meiting Wang

**Affiliations:** 1 School of Medical Engineering & Henan International Joint Laboratory of Neural Information Analysis and Drug Intelligent Design, Xinxiang Medical University, Xinxiang 453003, China; 2 School of Pharmacy, Xinxiang Medical University, Xinxiang 453003, China; 3 Shanghai Engineering Research Center of Molecular Therapeutics & New Drug Development, School of Chemistry and Molecular Engineering, East China Normal University, Shanghai 200062, China; 4 Faculty of Synthetic Biology, Shenzhen Institute of Advanced Technology, Chinese Academy of Sciences, Shenzhen 518055, China; 5 NYU-ECNU Center for Computational Chemistry at NYU Shanghai, Shanghai 200062, China; 6 Department of Chemistry, New York University, New York, NY 10003, USA; 7 Department of Theoretical Chemistry, Chemical Centre, Lund University, SE-221 00 Lund, Sweden

**Keywords:** LSD1 inhibitors, 3D-QSAR, molecular docking, molecular dynamics simulations

## Abstract

Lysine-specific demethylase 1 (LSD1) is a histone-modifying enzyme, which is a significant target for anticancer drug research. In this work, 40 reported tetrahydroquinoline-derivative inhibitors targeting LSD1 were studied to establish the three-dimensional quantitative structure–activity relationship (3D-QSAR). The established models CoMFA (Comparative Molecular Field Analysis (q^2^ = 0.778, $R_{\mathrm{pred}}^{2}$ = 0.709)) and CoMSIA (Comparative Molecular Similarity Index Analysis (q^2^ = 0.764, $R_{\mathrm{pred}}^{2}$ = 0.713)) yielded good statistical and predictive properties. Based on the corresponding contour maps, seven novel tetrahydroquinoline derivatives were designed. For more information, three of the compounds (**D1**, **D4**, and **Z17**) and the template molecule **18x** were explored with molecular dynamics simulations, binding free energy calculations by MM/PBSA method as well as the ADME (absorption, distribution, metabolism, and excretion) prediction. The results suggested that **D1**, **D4**, and **Z17** performed better than template molecule **18x** due to the introduction of the amino and hydrophobic groups, especially for the **D1** and **D4**, which will provide guidance for the design of LSD1 inhibitors.

## 1. Introduction

In 2004, the discovery of lysine specific demethylase 1 (LSD1) broke the previously held notion that histone lysine methylation was an irreversible process [1]. LSD1 (also known as KDM1A) can remove mono- or di-methylation of histones H3K4 and H3K9, which plays an important role in the regulation of histone modifications [2,3,4]. A series of recent studies have indicated that LSD1 can also affect the function of variety non-histone proteins, such as p53, DNA methyltransferase 1 (DNMT1), a signal transducer and activator of transcription 3 (STAT3), through removing mono- or di-methyl group [5,6,7]. Besides that, as a co-repressor of histone demethylase and transcription, LSD1 also plays a crucial role in gene expression, cell proliferation, and differentiation, which can lead to tumor development [8].

In the past few decades, many studies reported that overexpression of LSD1 was related to variety kinds of cancers [9,10,11,12,13,14]. Kahl et al. showed that overexpression of LSD1 was significantly associated with high a recurrence rate of prostate cancer [15], and Wang et al. found that LSD1 inhibited the invasion of breast cancer cells in vitro and metastasis of breast cancer cells in vivo [16]. In addition, LSD1 is also closely related to some high-risk cancers, such as liver cancer [17], lung cancer [18], and gastric cancer [19], etc. Inhibition of the overexpression of LSD1 could exert an anti-tumor effect. Therefore, LSD1 is a significant target for anticancer drug design [20].

**Figure 1:**
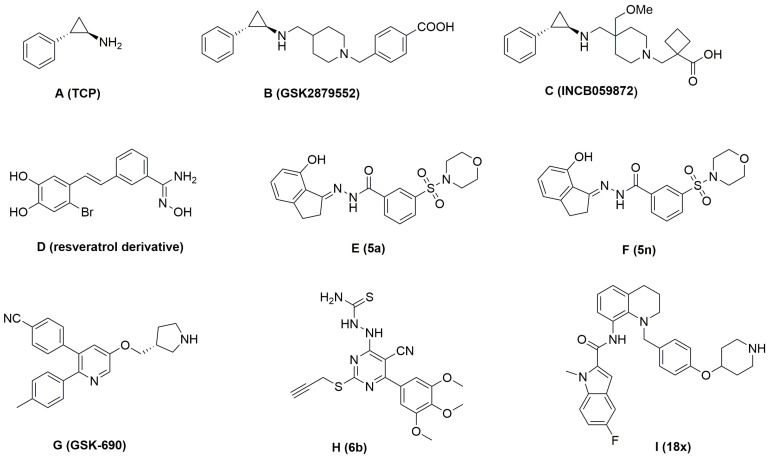
Structures of several reported LSD1 inhibitors.

Novel inhibitors targeting LSD1 have been continuously reported [21,22,23,24,25,26,27,28,29]. According to their mechanism of action, these inhibitors can be divided into two groups: reversible inhibitors and irreversible inhibitors. Irreversible inhibitors of LSD1 (Figure 1A–C) [25,26,30,31] developed rapidly and showed strong affinity with LSD1. However, partial irreversible inhibitors also caused some side effects in vivo because of the micromolar affinity with many targets. Compared with the irreversible inhibitors, reversible inhibitors have unique advantages in safety. Therefore, many kinds of reversible inhibitors targeting LSD1 have been promoted, as shown in Figure 1D–I [21,22,23,24,28,29]. Wang et al. designed and synthesized a series of reversible inhibitors targeting LSD1 based on tetrahydroquinoline derivatives, and these derivatives showed excellent inhibitory effect on LSD1 [29], such as the compound named **18x** (shown in Figure 1I), for which the value of IC_50_ is as high as 0.54 µM. Moreover, experiments reported that the compound **18x** also exhibited acceptable liver microsomal stability without significant toxic and side effects.

Due to the advantage of tetrahydroquinoline derivatives, the novel compounds that were created based on the tetrahydroquinoline derivatives should be highly potent. In this work, to keep the advantage of the derivatives, 40 reported tetrahydroquinoline derivative inhibitors were used to construct the three-dimensional quantitative conformational relationship (3D-QSAR) models, and then, seven novel tetrahydroquinoline derivatives with higher predicted activity were promoted. Among these seven novel compounds, three more promising molecules were chosen for further analysis. According to the results of docking, binding affinity calculation and ADME prediction, the selected three derivatives showed good bioavailability and drug-likeness.

## 2. Results and Discussion

### 2.1. CoMFA and CoMSIA Models

Based on the biological activity of the inhibitors, 3D-QSAR models of tetrahydroquinoline derivatives were developed with CoMFA and CoMSIA model. The results of the two models are listed in Table 1. It is generally considered that models with q^2^ > 0.5 have good internal validation ability [32], and those with $R_{\mathrm{pred}}^{2}$ > 0.6 have good external prediction ability [33]. As shown in Table 1, only CoMFA-S (modeled only with steric field), CoMFA-SE (modeled with both steric and electrostatic fields), and CoMSIA-SHDA satisfy these two conditions, which indicate that these three models have good internal validation and external prediction ability.

Compared with the CoMFA-SE model, CoMFA-S model shows better internal validation (q^2^ is 0.778) and external prediction ($R_{\mathrm{pred}}^{2}$ is 0.709) ability. In addition, the ONC, r^2^, SEE, and F-value of the CoMFA-S model are 2, 0.877, 0.336, and 96.151, respectively. The contributions of steric and electrostatic fields to the CoMFA-SE model are 60.1% and 39.9%, respectively, indicating that the steric field was the most important field in the model. Clearly, the CoMSIA-SHDA model has the best internal validation (q^2^ is 0.764) and external prediction ($R_{\mathrm{pred}}^{2}$ is 0.713) abilities among the six CoMSIA models. The values of ONC, r^2^, SEE, and F-value of the model are 7, 0.965, 0.198, and 86.831, respectively. The contributions of stereo, hydrophobic, hydrogen bond acceptor, and donor fields are 15%, 34.3%, 20.1%, and 30.7% respectively, suggesting that the hydrophobic and hydrogen bond donor fields play important roles in the model. Eventually, the CoMFA-S (later abbreviated as CoMFA) and the CoMSIA-SHDA (later abbreviated as CoMSIA) models were chosen as our final CoMFA and CoMSIA models, respectively. The predicted activity with CoMSA and CoMSIA models is shown in Supporting Information Table S1.

In Tropsha’s opinion [34], only the condition of $R_{\mathrm{pred}}^{2}$ > 0.6 cannot fully indicate that the established model has good external predictive ability. To evaluate the external predictive ability of these two models, some external predictive parameters were calculated [35], and the results are summarized in Table 2. A model with good external predictive power should satisfy the conditions (1, 2a or 2b, 3a or 3d, 4a or 4b, 5, and 6). Clearly, the CoMSIA model satisfies all the conditions. For the CoMFA model, condition 4a and other conditions are satisfied except for the condition 4b.

As shown in Table S1 (Supporting Information), the residual between the experimental and predicted values of CoMFA model is slightly larger, and therefore, the value of ${R^{\prime }}_{0}^{2}$ is slightly smaller. This is why condition 4b is not satisfied. In general, both the CoMFA and CoMSIA models developed by 3D-QSAR have good external prediction ability. It is worth noting that the CoMSIA model may be superior compared to the CoMFA model.

**Figure 2:**
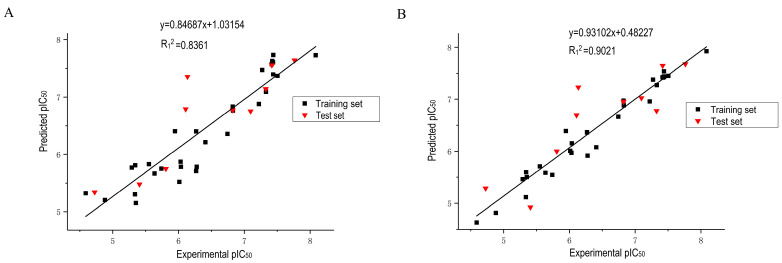
Scatter plots of experimental pIC_50_ values and predicted pIC_50_ values for CoMFA model (**A**) and CoMSIA model (**B**).

The scatter plots of the CoMSA and CoMSIA models are shown in Figure 2, with the *x*-axis is the experimental pIC_50_ value, and the *y*-axis is the predicted pIC_50_ value. From the figure, it can be seen that most of the points are distributed near the fitted line for these two models, indicating that the predicted pIC_50_ values match with the experimental values very well. Therefore, the linear correlation coefficient R_1_ of the CoMFA and CoMSIA model are 0.91 and 0.95, which also shows the reliability of these models.

Furthermore, the results of the Y-randomization test are summarized in Table 3. Obviously, the q^2^ and r^2^ values of the new models are very low, indicating that the previous model has good robustness.

### 2.2. CoMFA and CoMSIA Contour Maps

We used the StDev×Coeff function to display contour maps for each field, which can visualize the relationship between molecular structural features and biological activity. Among them, the visualization contributions of favorable and unfavorable regions were 80% and 20%, respectively.

**Figure 3:**
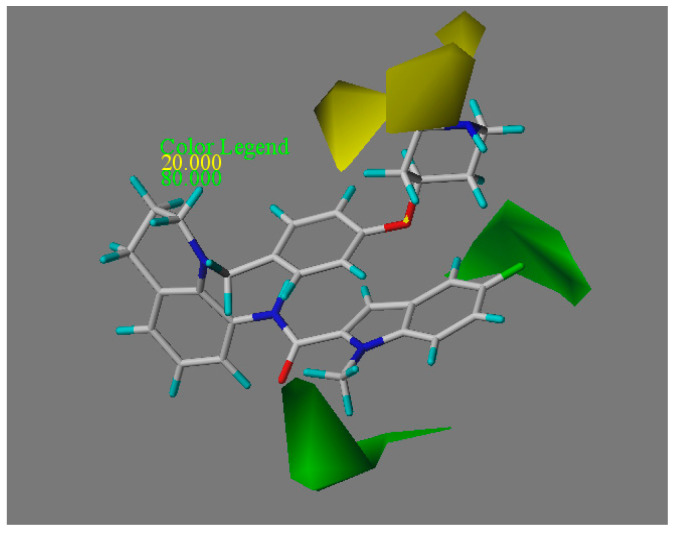
The CoMFA model contour map with compound **18x** as the reference.

The contour map of the CoMFA model is shown in Figure 3, with compound **18x** (compound **22**) as reference. In the CoMFA steric field, the green surfaces represent the addition of bulky groups here will be favorable for the biological activity, while the yellow surfaces mean bulky groups here may be unfavorable. There are two green surfaces near the R2 group, suggesting that the bulky group here would enhance the activity. Compound **18** (with two methyl groups at R2) has better activity than compound **19** (with two F atoms at R2), which also verifies the conclusion. On the other hand, there are two yellow surfaces near the R1 group, which suggests that the bulky group here would reduce the activity. This is confirmed by the following activity order: compound **3** (with a carbon chain at R1) > compound **4** (with a six-membered ring at R1) and compound **10** (with a five-membered ring at R1) > compound **12** (with a six-membered ring at R1).

**Figure 4:**
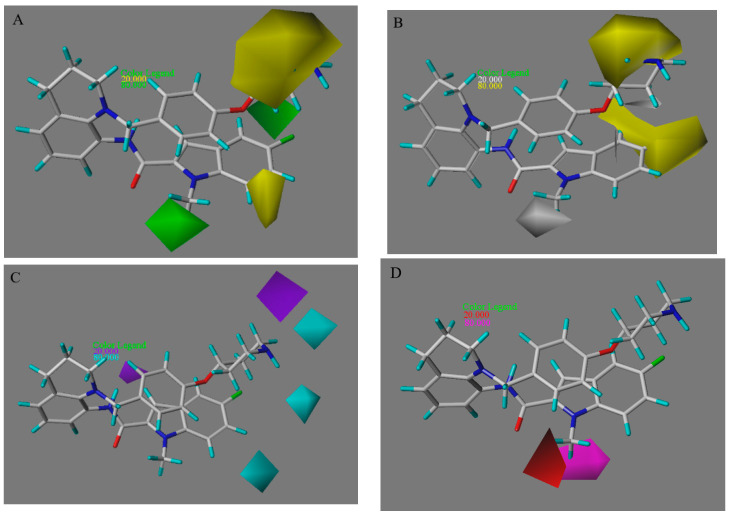
The CoMSIA model contour maps with compound **18x** as the reference. (**A**) The steric field. (**B**) The hydrophobic field. (**C**) The hydrogen bond donor field. (**D**) The hydrogen bond acceptor field.

The contour maps of the CoMSIA model are shown in Figure 4. The steric field contour map of CoMSIA (Figure 4A) was extremely similar to that of CoMFA. For the hydrophobic field (Figure 4B), a hydrophobic substitution would be favorable for the activity in the yellow surface and unfavorable for the activity in the white surface. Clearly, a hydrophobic group substitution near the R1 group is beneficial to the inhibitory activity, which is supported by the followed activity order: compound **3** (with an N atom at R1) > compound **11** (with an -NH_2_ group at R1) and compound **4** (with an N atom at R1) > compound **6** (with an -NH_2_ group at R1). Figure 4C shows the contour map of the hydrogen bond donor field for the CoMSIA model. The cyan surfaces represent that the hydrogen bond donor here is beneficial to the activity. Conversely, the purple surfaces suggest the hydrogen bond donor here will reduce the activity. For instance, at the R1 region of inhibitors, the inhibitory activity of compound **7** (pIC_50_ = 7.42389) > compound **12** (pIC_50_ = 7.27173); at the R2 region of inhibitors, the inhibitory activity of compound **21** (pIC_50_ = 6.26761) > compound **33** (pIC_50_ = 5.40671) and compound **36** (pIC_50_ = 5.33914) > compound **40** (pIC_50_ = 4.59108). Finally, for the hydrogen bond acceptor field (Figure 4D), the favorable and unfavorable surfaces of the hydrogen bond acceptor are colored magenta and red. For example, compound **13** used F atom towards the magenta surface instead of C atom on compound **16**, which can explain why compound **13** (pIC_50_ = 7.22185) is better than compound **16** (pIC_50_ = 6.82391). However, compound **24** is very similar to compound **13**, just adding an F atom towards the red surface, the pIC_50_ of which is decreased by 1.12 (pIC_50_ = 6.10791).

### 2.3. Design of New Derivatives

**Figure 5:**
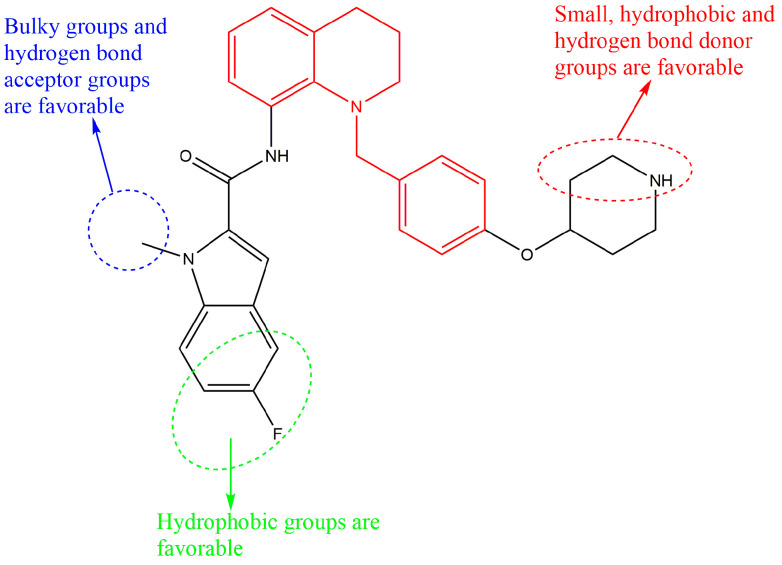
Structure-activity relationships (SAR).

The compound **18x** with IC_50_ = 0.54 µM, which exhibited superior drug properties, was selected as a template. The structure–activity relationship (SAR) information is summarized in Figure 5. As mentioned in Section 2.2, the introduction of small hydrophobic and hydrogen-bonding donor groups in the red region will enhance of inhibitor activity. Similarly, it is favorable to introduce appropriate hydrophobic groups in the green region. For the blue circle region, the bulky, hydrophilic, as well as hydrogen bonding acceptor group could be introduced. As mentioned before, the halogen elements (F and Cl atoms) and -NH_2_ groups were introduced into the red region, the F atom and methyl group were introduced into the green region, and the -NH_2_ and -OH groups were introduced into the blue region. Therefore, seven tetrahydroquinoline derivatives (**D1**, **D2**, **D4**, **Z5**, **Z17**, **P8**, and **P56**) were created.

The newly designed derivatives were docked into the pocket of LSD1 with Schrödinger, and the 2D diagrams of the interactions between the derivatives and LSD1 are depicted in the Supporting Information Figure S2. The results show that the introduced group -NH_2_ enhanced the interaction between the derivatives and LSD1, especially for hydrogen bonds between the derivatives and residue Asp555, Phe538, Glu559, and Pro808 of LSD1 (more details in the Supporting Information). These hydrogen bond interactions improved the binding stability of the complex. To export more details, molecular dynamic simulation and binding free energy calculation were performed for the complexes LSD1–D1, LSD1–D4, LSD1–Z17 and LSD1–18x. Actually, the structure, docking pose, docking score, and interaction of **Z17** are very similar to those of **18x**. Here, we selected **Z17** to verify the established model from another perspective.

The pIC_50_ values of these seven derivatives were predicted using the CoMFA and CoMSIA model, and the results are listed in Table 4. The values of pIC_50_ predicted with CoMFA and CoMSIA ranged from 6.41 to 6.94 and 7.19 to 8.39, respectively. Obviously, all of the seven derivatives, especially for D1 and D4, yielded a higher predicted pIC_50_ value than the template molecule **18x**. The docking scores, carried out with Schrödinger, are also listed in Table 4. The results show that all the seven newly designed derivatives report higher scores than **18x**, which is consistent with the both the predicted pIC_50_ and the calculated binding free energy (Section 2.5). It suggests that all designed compounds could have better inhibitory activity against LSD1.

### 2.4. MD Simulations Analyses

**Figure 6:**
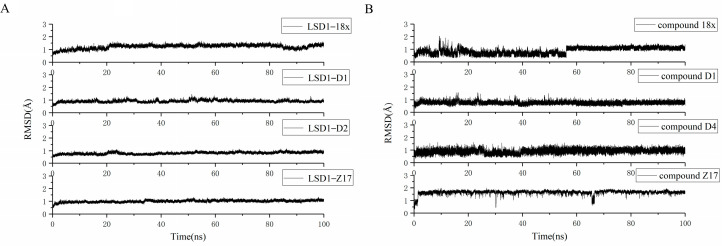
The RMSD results of the four systems. (**A**) The RMSD values of the complexes in the four systems. (**B**) The RMSD values of the ligands in the four systems.

In this work, the complexes LSD1–D1, LSD1–D4, LSD1–Z17, and LSD1–18x were utilized to perform for the molecular dynamics simulations of 100 ns. CPPTRAJ module [36] was employed to analyze the conformational stability of the complex system during the simulation by calculating the root-mean-square deviation (RMSD) for the Cα atom of the complex and the ligand, respectively. The RMSD values of the four systems are presented in Figure 6. It is worth noting that all the simulations were performed in triplicates, and the results of other simulations are depicted in the Supporting Information Figure S3. Clearly, for each system, the complex was stable during the MD simulation process, and the RMSD values of both the complex and ligand were less than 2 Å.

**Figure 7:**
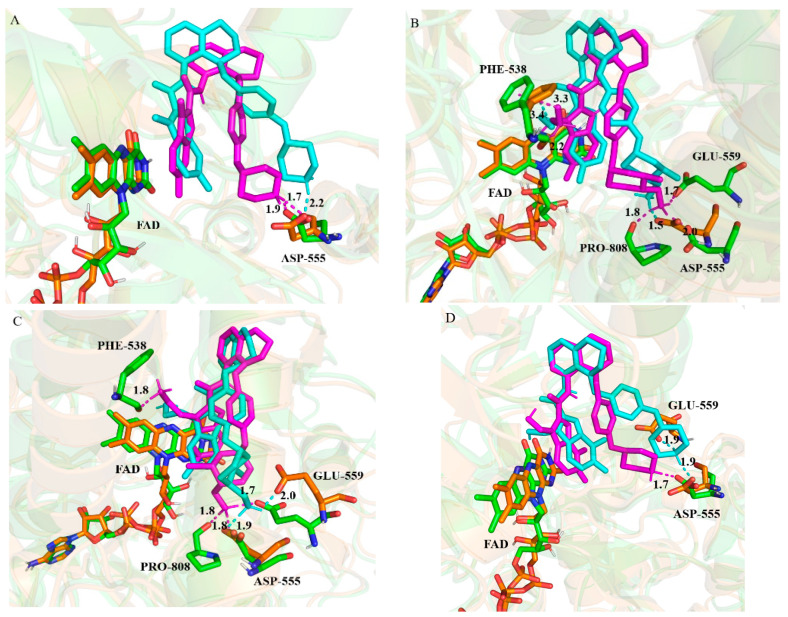
The superposition of the docking structures and MD average structures of complexes LSD1–18x (**A**), LSD1–D1 (**B**), LSD1–D4 (**C**), and LSD1–Z17 (**D**). The key residues and compounds of molecular docking structure are in orange and cyan, respectively. The key residues and compounds of the average structures are shown in green and magenta.

Figure 7 indicates that the superposition of the docking structure and MD average structure during MD equilibrium stage for each system. It was worth noting that the four compounds are still located well in the substrate binding region, which share the same binding mode with a “U-shaped” conformation. Furthermore, the hydrophobic and polar interactions between the compound and surrounding residues are favorable for maintaining the stability of the complex. Amazingly, as shown in Table 5, the hydrogen bonds between the four compounds and the residue Asp555 as well as the arene–cationic interaction between the compound **D1** and Phe538 were stable during the MD simulations. Compared with LSD1–18x, during the simulations of LSD1–D1 and LSD1–D4, the hydrogen bonds Asp555=O⋯HN were kept, and the bond energies were almost the same (−13.2 kcal/mol, −13.7 kcal/mol versus −13.3 kcal/mol). Furthermore, due to the introduced -NH_2_ group, two more stronger H-bonds, namely Glu559=O⋯HN and Pro808=O⋯HN, with a bond energy of −20.1 kcal/mol, −13.3 kcal/mol and −18.2 kcal/mol, −5.8 kcal/mol, were formed for LSD1–D1 and LSD1–D4. However, during the simulation of LSD1–Z17, the H-bond Glu559=O⋯HN disappeared, but the newly formed H-bond Asp555-HO⋯HN was stronger than that of LSD1–18x, with a value of −13.1 kcal/mol. In addition, the last 20 ns of the production trajectory were used to calculate the H-bonds occupancy, and the results are also depicted in Table 5. Most of the H-bonds’ occupancies were over 80%, especially for the mentioned H-bonds of the residues Asp555, Pro888, and Glu559.

Based on the analysis, it could be found that the residue Asp555 played a crucial role during the binding of the compounds to LSD1. Compared to the compound **18x**, the -NH_2_ groups of compounds **D1** and **D4** at R1 and R2 regions not only formed hydrogen bonds with Asp555, but more importantly, they also formed hydrogen bonds with Phe538, Glu559, and Pro808. In contrast, the -OH group of the compound **Z17** at R2 region did not form any interactions with these residues. This suggests that the introduction of the -NH_2_ group is more effective than the -OH group in this study, which will provide some guidance for the design of LSD1 inhibitors in the future.

### 2.5. Binding Free Energy Calculation

In order to predict the binding affinity of the four compounds with LSD1, the MM/PBSA method was utilized to calculate the binding free energy. MM/PBSA was performed for all the three trajectories, and the average results are summarized in Table 6 (more detail listed in Tables S2–S4). As shown in Table 6, the $G_{\mathrm{bind}}$ of complexes LSD1–D1, LSD1–D4, LSD1–Z17, and LSD1–18x are −55.29 kcal/mol, −43.93 kcal/mol, −30.09 kcal/mol, and −29.45 kcal/mol, respectively. It suggests that these three novel compounds may inhibit the activity of LSD1 better than compound **18x**, which is consistent with predicted pIC_50_ and the docking score. In addition, we also analyzed contribution of van der Waal and electrostatic interaction energy for each command. As listed in Table 6, electrostatic energy makes a prominent contribution to the binding free energy of all systems, which indicates that electrostatic interactions between the compound and the LSD1 play a key role. A possible reason may be due to the interaction between the basic N on the six-membered ring or amino group of the compound and the negatively charged amino acids Asp555 as well as Glu559. Complexes LSD1–D1 and LSD1–D4 are much the same. In addition, the van der Waals forces between the ligand and the receptor contribute to the stability of the complexes. However, the positive value of the polar solvation energy ($G_{\mathrm{pol}}$) indicates that it is not favorable for the receptor–ligand binding. Conversely, the nonpolar solvation energy ($G_{\mathrm{np}}$) favors the binding free energy.

**Figure 8:**
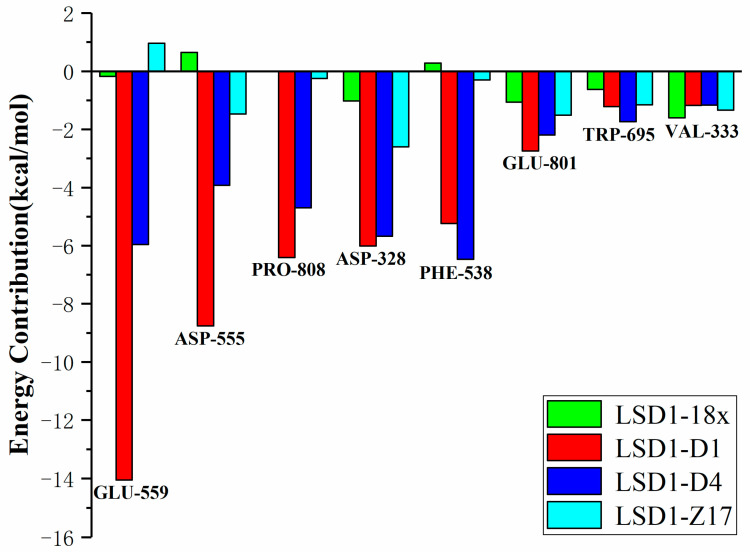
Binding free energy decomposition plot.

Energy decomposition was carried out to illuminate the weightiness of individual residues in the binding process of the compound to LSD1 (shown in Figure 8). The energy contributions of the most contributive residues (Glu559, Asp555, Pro808, Asp328, Phe538, Glu801, Trp695, and Val333) were summarized in Figure 8. Clearly, compared to complexes LSD1–18x and LSD1–Z17, the residues Glu559, Asp555, Pro808 and Phe538 had better energy contributions to complexes LSD1–D1 and LSD1–D4. This is because LSD1 formed the hydrogen bonds and salt bridges with the introduced groups (-NH_2_) in the complexes LSD1–D1 and LSD1–D4. Furthermore, the introduction of hydrophobic groups made the compounds more stably bound in the hydrophobic pocket, which consisted of Val333, Phe538, Trp695, and Pro808. The decomposition of binding free energy suggests that Phe538, Asp555, Glu559, and Pro808 might be the key residues in the ligand–receptor binding process, and the hydrophobic interactions are also essential.

### 2.6. ADME and Bioavailability Analysis

To evaluate the pharmacokinetic properties of these seven newly designed derivatives and the compound **18x**, ADME analysis was also performed (Listed in Table 7). For the bioavailability, the results of molecular weight (MW), saturation (Csp^3^), number of rotatable bonds, and topological polar surface area (TPSA) are all within the optimal range for these seven compounds except for the number of rotatable bonds (N) of compounds **D2**, **P8** and **P56**. Moreover, as shown in Table 7, all the pharmacokinetic properties are good except for BBB. All the predicted lipophilicity (log P) and solubility (log S) are also within the optimal range. Compared with **18x**, **D1** is more lipophilic, while **D2** and **D4** are more hydrophilic. The results of HIA and drug-likeness also suggest these derivatives have high gastrointestinal absorption ability and drug-like properties. The result of ${\mathrm{logK}}_{p}$ shows that these seven compounds are able to maintain skin permeability. Moreover, the compounds **D1**, **D2**, and **D4** also show inhibitory activity against CYP3A4. This means they could be eliminated by human metabolism. Taking the predicted values of pIC_50_ (Table 4) and the calculated binding free energies (Table 6) into consideration, these newly designed derivatives should have high bioavailability and excellent drug-like properties, especially for **D1** and **D4**.

## 3. Materials and Methods

### 3.1. Data Sets and Structure Alignment

Forty reported tetrahydroquinoline derivative inhibitors were used to establish 3D-QSAR models. The structures and biological activities are shown in Table 8. The IC_50_ (range from 0.008–25.64 μM) for compounds represent semi-inhibitory concentration values, which cannot be used directly with 3D-QSAR studies. Therefore, IC_50_ was converted into pIC_50_ (−log IC_50_), and the corresponding values range from 4.591 to 8.084.

**Figure 9:**
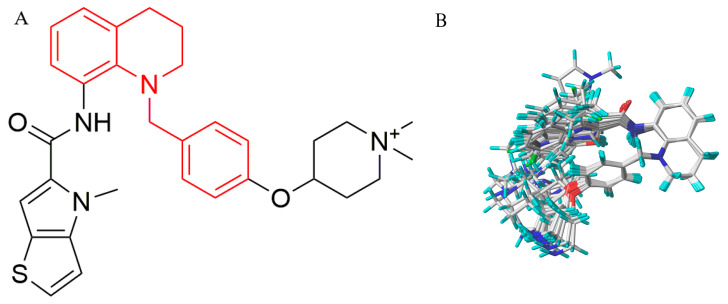
(**A**) That marked in red is the common skeleton of the compounds and (**B**) the alignment result of the training set.

The 3D structures of all compounds were constructed in SYBYL-X2.0 firstly (2.0, Tripos International, St. Louis, MS, USA) and then optimized by Tripos standard force fields [37] together with Gasteiger–Huckel charges [38]. For the minimization, Powell gradient algorithm was applied. The maximum number of iterations was set to 1000, and the energy gradient convergence criterion was 0.001 kcal/(mol×Å). Generally, the training sets and test sets should meet the following conditions: (I) the pIC_50_ values of the training set should satisfy the maximum value (test) ≤ maximum value (training) and minimum value (test) ≥ min (training) [39]; (II) the number of training sets accounts for 75–80% and the number of test sets accounts for 20–25% [27]. Consequently, 75% of the 40 compounds (30 compounds) were randomly assigned to the training set, and the test set was composed of the remaining ten molecules. For the establishment of the 3D-QSAR model, one of the primary steps is the selection of the template skeleton. The lowest-energy conformation of the most active molecule (inhibitor 1) was selected as the template to construct and optimize other molecules. The common backbone and the alignment of the training set are shown in Figure 9A and Figure 9B, respectively.

### 3.2. 3D-QSAR Models and Statistical Analysis

In this study, both comparative molecular field analysis (CoMFA) [40,41] and comparative molecular similarity index analysis (CoMSIA) [42] were used to build 3D-QSAR models. CoMFA was applied to characterize the relationship between the steric and electrostatic fields around the ligand and the biological activity of the ligand. For the CoMSIA model, besides electrostatic and steric fields, it also analyzed the hydrophobic, hydrogen bond acceptor, and donor fields. More importantly, a distance-dependent Gaussian function was introduced into the CoMSIA mothed for the calculation of the interaction between probe atoms or groups and molecules [43], which effectively avoided the defects caused by the functional forms of electrostatic and steric fields in the conventional CoMFA method.

The partial least squares (PLS) regression method was employed to analyzed the CoMFA and CoMSIA models [44]. The statistical indicators like predicted residual sum of squares (PRESS) and the cross-validation correlation coefficient were used to evaluate the predictive power of the models. Leave-one-out (LOO) was utilized to obtain the cross-validation coefficient q^2^ and the optimal number of components (ONC) [45]. The statistical index PRESS and q^2^ could be calculated by the following formulas [46]:

(1) $$\mathrm{PRESS}=\sum {\left(Y_{p}{-Y}_{a}\right)}^{2}$$

(2) $$\mathrm{TSS}=\sum {\left(Y_{a}{-\bar{Y}}_{a}\right)}^{2}$$

(3) $$q^{2}=1-\frac{\mathrm{PRESS}}{\mathrm{TSS}}$$

where $Y_{a}$ and $Y_{p}$ represent the experimental pIC_50_ value and predicted pIC_50_ value of the compounds in the test set, respectively, and ${\bar{Y}}_{a}$ expresses the average of the whole training set. It is worth noting that the proposed model is statistically significant only when q^2^ > 0.5. Then, with non-cross-validation, we can obtain the non-cross-validation correlation coefficient (r^2^), the F-statistic value (F), the standard error of estimate (SEE), and the contributions of the individual fields in the model. The predictive ability of the model is evaluated by calculating the predictive correlation coefficient ($R_{\mathrm{pred}}^{2}$), which is calculated as follows [47]:

(4) $$R_{\mathrm{pred}}^{2}=\frac{\mathrm{SD}-\mathrm{PRESS}}{\mathrm{SD}}$$

where SD is the sum of squared deviations of each activity value in the test set from the mean value of the activity values in the training set. The closer the $R_{\mathrm{pred}}^{2}$ is to 1, the stronger the predictive ability of the model.

In addition to these internal parameter validations, we also need a series of external validation coefficients such as R^2^, k, k′, $R_{0}^{2}$, ${R\prime }_{0}^{2}$, and $r_{m}^{2}$, to further assess the predictive performance of the model built by 3D-QSAR, where R^2^ represents the correlation coefficient between the experimental activity value in the test set and the activity value predicted by the model. $R_{0}^{2}$ and k stand for the correlation coefficient and linear slope between experimental activity values as independent variables (X) and predicted activity values as dependent variables (Y) in the test set, respectively. ${R\prime }_{0}^{2}$ and k′ are the correlation coefficient and linear slope between predicted activity values as independent variables (X) and experimental activity values as dependent variables (Y) in the test set, respectively. $r_{m}^{2}$ represents the approximation between the experimental activity value and the predicted value in the test set. The following are the calculation formulas of these parameters [48]:

(5) $$R^{2}=\frac{{\left[\sum \left(Y_{a}{-\bar{Y}}_{a}\right)\left(Y_{p}{-\bar{Y}}_{p}\right)\right]}^{2}}{\sum {\left(Y_{p}{-\bar{Y}}_{p}\right)}^{2}\times \sum {\left(Y_{a}{-\bar{Y}}_{a}\right)}^{2}}$$

(6) $$k=\frac{\sum \left(Y_{a}{\times Y}_{p}\right)}{\sum {\left(Y_{p}\right)}^{2}}$$

(7) $$k\prime =\frac{\sum \left(Y_{a}{\times Y}_{p}\right)}{\sum {\left(Y_{a}\right)}^{2}}$$

(8) $$R_{0}^{2}=1-\frac{\sum {\left(Y_{a}{-k\times Y}_{p}\right)}^{2}}{\sum {\left(Y_{a}{-\bar{Y}}_{a}\right)}^{2}}$$

(9) $${R\prime }_{0}^{2}=1-\frac{\sum {\left(Y_{p}{-k\prime \times Y}_{a}\right)}^{2}}{\sum {\left(Y_{p}-{\bar{Y}}_{p}\right)}^{2}}$$

(10) $$r_{m}^{2}{=R}^{2}\times \left(1-\sqrt{R^{2}-R_{0}^{2}}\right)$$

where ${\bar{Y}}_{a}$ and ${\bar{Y}}_{p}$ are the average values corresponding to $Y_{a}$ and $Y_{p}$.

Finally, the Y-randomization test was applied to test and verify the stability of the 3D-QSAR model [49]. By keeping the independent variable X constant and shuffling the dependent variable randomly 10 times, the q^2^ and r^2^ of the new models are recalculated. If the values of q^2^ and r^2^ are very low, the robustness of the established model can be indicated.

### 3.3. Molecular Docking

To study the interaction between newly designed derivatives and LSD1, we applied the Glide module in Maestro [50,51,52] for molecular docking. To be consistent with the Wang’s work [29], we used the same structure (the X-ray cocrystal structure of substrate molecule with LSD1 can be found in Supporting Information Figure S1, PDB code: 5LHI), which was obtained from RCSB PDB (https://www.rcsb.org/ accessed on 1 October 2021). The downloaded protein was subjected to the Protein Preparation Wizard module in Maestro for structural optimization, including hydrogenation, dehydration, protonation, and energy minimization. Similarly, the 2D structures of ligands created with MarvinSketch were imported into the Ligprep module for optimization and the generation of multiple different conformations. Afterwards, a docking box with size 20 Å × 20 Å × 20 Å was generated with the substrate binding domain as the docking site. Finally, in the Glide module, the optimized ligands were docked to the substrate binding site. The docking precision was set as SP (standard precision), and the binding poses with the top ten Glide score were selected. According to the scoring results and the superposition with ligand in the substrate region of the crystal structure (5LHI), the final docking conformation was selected for further study.

### 3.4. Molecular Dynamics Simulation

The molecular dynamics (MD) of the complexes LSD1-D1, LSD1-D4, LSD1-Z17, and LSD1-18x were carried out with AMBER18 package [53]. Table 4 showed the structures of compounds D1, D4, and Z17, with the highest docking score was taken as the initial conformation of the complex. For the protein, ff14SB force field [54] was applied. The ligands were described with the general AMBER force field (GAFF) [55]. Each complex was solvated in a cubic periodic boundary box of TIP3P molecules extending at 12 Å from the ligand. Chloride ions were randomly added to the simulated system to maintain electrical neutrality [56].

Each complex was subjected to a 2500 steps of minimization, followed by 250 ps heating and 50 ps equilibration. Finally, a 100 ns production simulation was performed at constant pressure (1 atm) and constant temperature (300 K). All bonds involving hydrogen atoms were constrained by adopting the SHAKE algorithm, allowing for a 2 fs time step [57]. Particle mesh Ewald (PME) [58] and periodic boundary condition were used to treat the electrostatic interactions. The cut-off of Lennard–Jones interaction was set to 10 Å.

### 3.5. Binding Free Energy Calculation

In this work, the binding affinity of the protein and the molecules were predicted with the widely used method Molecular Mechanics/Poisson Boltzmann Surface Area (MM/PBSA) [59]. With this method, the combined free energy was divided into molecular mechanics terms and solvation energy. When using MM/PBSA, the binding free energy is given by

(11) $$G_{\mathrm{bind}}{=E}_{\mathrm{bond}}{+E}_{\mathrm{ele}}{+E}_{\mathrm{vdW}}{+G}_{\mathrm{pol}}{+G}_{\mathrm{np}}-\mathrm{TS}$$

where $G_{\mathrm{bind}}$ is the final binding free energy. $E_{\mathrm{bond}}$ denotes the internal energy caused by the bond, angle, and dihedral angle terms in the molecular mechanical (MM) force field. In the single-track method, this term is always equal to zero. $E_{\mathrm{ele}}$ and $E_{\mathrm{vdw}}$ represent electrostatic energy calculated by MM force field and van der Waals contribution, respectively. The polar contribution $G_{\mathrm{pol}}$ is obtained by solving the PB equation, and the non-polar contribution $G_{\mathrm{np}}$ is estimated by linear relationship with the solvent-accessible surface area (SASA). In addition, TS (absolute temperature T multiplied by the entropy S) is known as the entropy contribution [60]. Considering the huge computational effort required for the calculation of this value and its small impact on the results [27,61,62,63], we neglected this part of the calculation in this work. In this work, 1000 frames from the last 20 ns of the simulation were used to calculate the free energy difference, and the results were carried out with the mmpbsa.py program [64].

### 3.6. ADME Prediction

In order to evaluate the drug-likeness of the newly designed derivatives, the SwissADME service station [65] was plied to perform the drug absorption, distribution, metabolism, and excretion (ADME) analysis. The evaluation indicators include bioavailability evaluations such as molecular weight, lipophilicity [66], saturation [67], and polarity [68] as well as human intestinal absorption (HIA) [69], blood–brain barrier (BBB) penetration [70], cytochrome P450-3A4 (CYP3A4) enzyme inhibition [71,72], skin permeability ($\log K_{p}$) [73], etc.

## 4. Conclusions

In this study, we collected 40 tetrahydroquinoline derivatives as LSD1 reversible inhibitors to establish a 3D-QSAR model. Through a series of statistical tests, the developed models CoMFA and CoMSIA reported good statistical and predictive properties with q^2^ = 0.778, $R_{\mathrm{pred}}^{2}$ = 0.709 and q^2^ = 0.764, $R_{\mathrm{pred}}^{2}$ = 0.713, respectively. The docking results suggested all of the seven newly designed derivatives report higher scores than the template molecule **18x**. Considering the molecular dynamics simulation and activity prediction, the two compounds **D1** and **D4** also showed better results than template molecule **18x**. The conclusion was further verified by the binding free energy calculation. In addition, the introduction of -NH_2_ groups enhanced the interaction between the derivatives **D1**, **D4**, and the residues Phe538, Glu559, as well as Pro808, which improved the binding stability of the LSD1 and the derivatives. Moreover, ADME prediction and bioavailability analysis also indicated that **D1** and **D4** had high bioavailability and excellent drug-like properties. We hope that this study can provide powerful reference for the design of LSD1 inhibitors in the future.

## Figures and Tables

**Table 1:** Statistical parameters of CoMFA and CoMSIA model. (S, steric; E, electrostatic; H, hydrophobic; A, H-bond acceptor; D, H-bond donor).

	q^2^	ONC	r^2^	R^2^_pred_	SEE	F-Value	Contributions				
							S	E	H	A	D
CoMFA- S	0.778	2	0.877	0.709	0.336	96.151	1				
CoMFA- E	0.417	3	0.873	0.037	0.347	59.619		1			
CoMFA- SE	0.709	3	0.914	0.600	0.287	91.530	0.601	0.399			
CoMSIA- EHDA	0.666	6	0.966	0.322	0.190	110.135		0.359	0.222	0.155	0.264
CoMSIA- SHDA	0.764	7	0.965	0.713	0.198	86.831	0.150		0.343	0.201	0.307
CoMSIA- SEDA	0.661	6	0.959	0.281	0.209	90.663	0.134	0.419		0.176	0.271
CoMSIA- SEHD	0.719	4	0.960	0.498	0.198	151.613	0.127	0.393	0.237		0.243
CoMSIA- SEHA	0.722	6	0.967	0.451	0.190	110.811	0.147	0.425	0.267	0.162	
CoMSIA- ALL	0.705	6	0.970	0.423	0.179	124.592	0.102	0.331	0.195	0.144	0.228

**Table 2:** The external verification calculation results of the CoMFA and CoMSIA models.

Condition	Parameters	Threshold Value	CoMFA	CoMSIA
1	$R^{2}$	>0.6	0.754	0.749
2a	$R_{0}^{2}$	$\mathrm{Close}\;\mathrm{to}\;\mathrm{value}\;\mathrm{of}\;R^{2}$	0.752	0.745
2b	${R^{\prime }}_{0}^{2}$	$\mathrm{Close}\;\mathrm{to}\;\mathrm{value}\;\mathrm{of}\;R^{2}$	0.650	0.706
3a	k	0.85 < k < 1.15	0.971	0.975
3b	$k^{\prime }$	$0.85\;<\;k^{\prime }$ < 1.15	1.025	1.021
4a	$(R^{2}$ $-R_{0}^{2}$ $)/R^{2}$	<0.1	0.003	0.005
4b	$(R^{2}-$ ${R^{\prime }}_{0}^{2}$ $)/R^{2}$	<0.1	0.138	0.057
5	$\left|R_{0}^{2}-{R^{\prime }}_{0}^{2}\right|$	<0.3	0.102	0.039
6	$r_{m}^{2}$	>0.5	0.720	0.702

**Table 3:** The results of Y-randomization validation.

	CoMFA		CoMSIA	
Iteration	q^2^	r^2^	q^2^	r^2^
Random_1	−0.116	0.111	−0.117	0.161
Random_2	−0.098	0.118	−0.097	0.424
Random_3	−0.174	0.326	−0.159	0.173
Random_4	−0.094	0.120	−0.018	0.248
Random_5	−0.026	0.149	−0.007	0.156
Random_6	−0.046	0.181	−0.03	0.471
Random_7	−0.113	0.129	−0.142	0.247
Random_8	−0.209	0.478	0.007	0.613
Random_9	−0.34	0.281	−0.207	0.095
Random_10	−0.18	0.144	−0.071	0.279

**Table 4:** The structure, predicted activity and docking score of the newly designed derivatives.

No.	R1	R2	Predicted pIC_50_		Glide Score (kcal/mol)
			CoMFA	CoMSIA	
**18x**	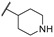	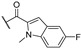	6.40	6.37	−6.23
**D1**	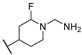	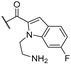	6.74	8.21	−10.20
**D2**	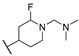	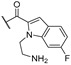	6.94	8.09	−8.51
**D4**	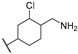	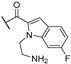	6.41	8.39	−9.32
**Z5**	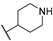	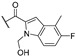	6.79	7.19	−8.58
**Z17**	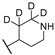	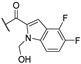	6.58	7.29	−8.09
**P8**	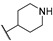	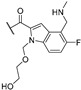	6.56	7.78	−8.71
**P56**	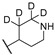	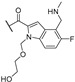	6.51	7.91	−7.49

**Table 5:** Hydrogen bonds of each complex.

Complex	Docking			MD			
	H-Bond	Length (Å)	Energy (kcal/mol)	H-Bond	Length (Å)	Energy (kcal/mol)	Hydrogen Bond Occupancy
LSD1–18x	Asp555=O⋯HN	1.7	−9.0	Asp555=O⋯HN	1.9	−13.3	50%
				Asp555–HO⋯HN	2.2	−5.5	80%
LSD1–D1	Asp555–HO⋯HN	1.5	−6.7	Asp555=O⋯HN	2.0	−13.2	45%
				Glu559=O⋯HN	1.7	−20.1	100%
				Pro808=O⋯HN	1.8	−13.3	100%
				Phe538=O⋯HN	2.2	−1.1	20%
LSD1–D4	Asp555–HO⋯HN	1.8	−5.0	Asp555=O⋯HN	1.9	−13.7	100%
				Glu559=O⋯HN	1.7	−18.2	100%
				Pro808=O⋯HN	1.8	−5.8	100%
				Phe538=O⋯HN	1.8	−5.7	65%
LSD1–Z17	Asp555=O⋯HN	1.9	−2.8	Asp555–HO⋯HN	1.7	−13.1	80%
	Glu559=O⋯HN	1.9	−12.5				

**Table 6:** Binding free energies of protein–ligand complexes (kcal/mol).

Contribution	LSD1–D1	LSD1–D4	LSD1–Z17	LSD1–18x
$E_{\mathrm{vdW}}$	−44.19	−56.09	−56.49	−46.43
$E_{\mathrm{ele}}$	−500.90	−280.71	−164.01	−161.03
$G_{\mathrm{pol}}$	459.32	298.54	195.77	183.03
$G_{\mathrm{np}}$	−5.52	−5.67	−5.35	−5.03
$G_{\mathrm{bind}}$	−55.29	−43.93	−30.09	−29.45
pIC_50_^a^	8.21	8.09	7.29	6.37
				6.27 ^b^

^a^ Predicted with CoMSIA model. ^b^ experimental value.

**Table 7:** Bioavailability and pharmacokinetics prediction.

No.	MW (g mol^−1^)	Fraction Csp^3^	N	TPSA (Å^2^)	Log P	Log S	HIA	BBB	CYP3A4 Inhibition	Log K_p_ (cm s^−1^)	Drug-Likeness Lipinski
**18x**	512.62	0.32	7	58.53	4.83	−6.35	High	Yes	No	−5.61	Yes
**D1**	588.69	0.36	10	101.78	4.11	−6.03	High	No	Yes	−6.70	Yes
**D2**	616.74	0.40	11	79.00	4.75	−6.74	High	No	Yes	−6.17	Yes
**D4**	604.16	0.38	10	98.54	4.9	−6.52	High	No	Yes	−6.34	Yes
**Z5**	542.64	0.34	8	78.76	3.78	−6.27	High	No	No	−6.00	Yes
**Z17**	550.63	0.32	8	78.76	3.59	−6.15	High	No	No	−6.24	Yes
**P8**	615.74	0.40	13	100.02	4.68	−5.63	High	No	No	−7.26	Yes
**P56**	619.76	0.40	13	100.02	4.64	−5.65	High	No	No	−7.28	Yes
Optimal range	<800	0.25–1	≤10	20–130	−0.7–5	−10–6	-	-	-	-	-

**Table 8:** Structures and inhibitory activity of tetrahydroquinoline derivatives.

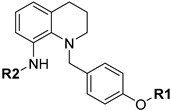				
No.	Chemical Structures		Inhibitory Activity	
	R1	R2	IC_50_ (μM)	pIC_50_
1	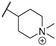	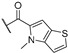	0.00825	8.08355
2 ^b^	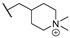	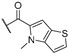	0.01726	7.76296
3	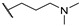	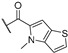	0.03126	7.50501
4	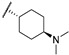	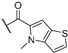	0.03626	7.44057
5	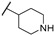	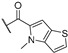	0.03637	7.43926
6	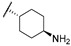	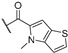	0.03658	7.43676
7	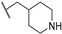	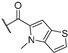	0.03768	7.42389
8 ^b^	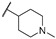	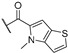	0.03825	7.41737
9	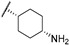	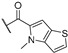	0.03834	7.41635
10	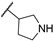	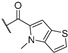	0.04678	7.32994
11 ^b^	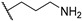	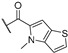	0.04736	7.32459
12	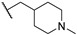	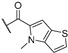	0.05349	7.27173
13	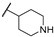	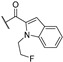	0.06000	7.22185
14 ^b^	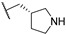	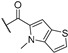	0.08035	7.09501
15	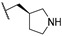	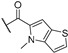	0.14856	6.82810
16	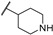	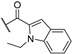	0.15000	6.82391
17 ^b^	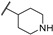	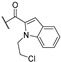	0.15000	6.82391
18	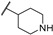	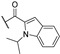	0.18000	6.74473
19	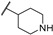	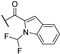	0.39000	6.40894
20	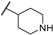	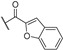	0.53000	6.27572
21	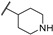	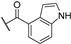	0.54000	6.26761
22 ^a^	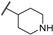	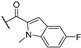	0.54000	6.26761
23 ^b^	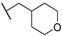	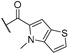	0.73232	6.13530
24 ^b^	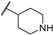	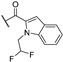	0.78000	6.10791
25	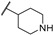	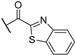	0.92000	6.03621
26	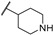	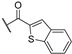	0.93000	6.03152
27	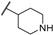	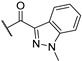	0.97000	6.01323
28	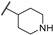	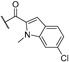	1.13000	5.94692
29 ^b^	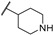	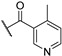	1.56000	5.80688
30	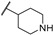	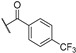	1.82000	5.73993
31	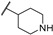	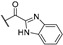	2.31000	5.63639
32	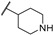	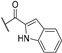	2.81000	5.55129
33 ^b^	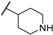	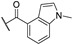	3.92000	5.40671
34	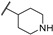	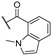	4.44000	5.35262
35	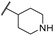	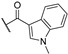	4.55000	5.34199
36	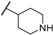	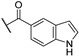	4.58000	5.33914
37	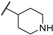	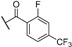	5.12000	5.29073
38	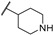	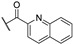	13.0900	4.88306
39 ^b^	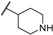	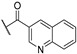	18.8000	4.72584
40	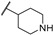	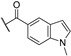	25.6400	4.59108

^a^ Also named as **18x**. ^b^ The compounds in the test set.

## Data Availability

Not applicable.

## Supplementary Materials

The following supporting information can be downloaded at: https://www.mdpi.com/article/10.3390/molecules27238358/s1, Figure S1: X-ray cocrystal structure of substrate molecule with LSD1 (PDB code: 5LHI); Figure S2: 2D diagrams of the interactions between the compounds and LSD1 (PDB code: 5LHI); Figure S3: The RMSD results for the second simulation and the third simulation; Table S1: Predicted activity results with CoMFA and CoMSIA models; Table S2: Binding free energies of protein–ligand complexes from the first MD simulations. All the energies are in kcal/mol; Table S3: Binding free energies of protein–ligand complexes from the second MD simulations. All the energies are in kcal/mol; Table S4: Binding free energies of protein–ligand complexes from the third MD simulations. All the energies are in kcal/mol.
