# Supplementary material for: Design Two Novel Tetrahydroquinoline Derivatives against Anticancer Target LSD1 with 3D-QSAR Model and Molecular Simulation
Source: Molecules. 2022 Nov 30;27(23):8358. doi: 10.3390/molecules27238358 (PMC9739212; doi:10.3390/molecules27238358)
Supplement: Supplementary file 1 [file molecules-27-08358-s001.zip › molecules-2017737-supplementary.pdf]

Design two novel tetrahydroquinoline derivatives against anticancer target LSD1 with 3D-QSAR model and molecular simulation.

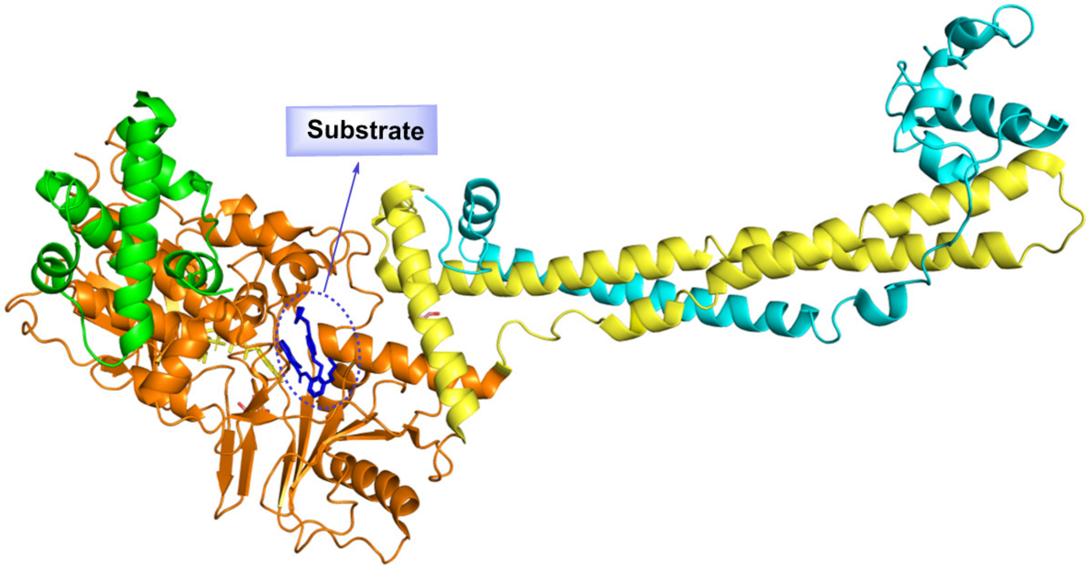

Figure S1. X-ray cocrystal structure of substrate molecule with LSD1 (PDB code: 5LHI).

Table S1. Predicted activity results with CoMFA and CoMSIA models.

No.	Actual pIC_{50}	CoMFA		CoMSIA	
		Predicted pIC_{50}	Residual	Predicted pIC_{50}	Residual
1	8.084	7.729	0.355	7.924	0.16
2	7.763	7.642	0.121	7.678	0.085
3	7.505	7.369	0.136	7.451	0.054
4	7.441	7.732	-0.291	7.43	0.011
5	7.439	7.394	0.045	7.538	-0.099
6	7.437	7.604	-0.167	7.441	-0.004
7	7.424	7.629	-0.205	7.418	0.006
8	7.417	7.557	-0.14	7.646	-0.229
9	7.416	7.572	-0.156	7.425	-0.009
10	7.33	7.092	0.238	7.27	0.06
11	7.325	7.141	0.184	6.776	0.549
12	7.272	7.472	-0.2	7.378	-0.106
13	7.222	6.88	0.342	6.955	0.267
14	7.095	6.753	0.342	7.02	0.075
15	6.828	6.765	0.063	6.88	-0.052
16	6.824	6.836	-0.012	6.97	-0.146

17	6.824	6.774	0.05	6.952	-0.128
18	6.745	6.358	0.387	6.665	0.08
19	6.409	6.215	0.194	6.077	0.332
20	6.276	5.783	0.493	5.916	0.36
21	6.268	5.713	0.555	6.36	-0.092
22	6.268	6.403	-0.135	6.367	-0.099
23	6.135	7.355	-1.22	7.228	-1.093
24	6.108	6.792	-0.684	6.693	-0.585
25	6.036	5.783	0.253	6.154	-0.118
26	6.032	5.872	0.16	5.97	0.062
27	6.013	5.523	0.49	6.008	0.005
28	5.947	6.404	-0.457	6.389	-0.442
29	5.807	5.749	0.058	6	-0.193
30	5.74	5.751	-0.011	5.545	0.195
31	5.636	5.668	-0.032	5.587	0.049
32	5.551	5.829	-0.278	5.708	-0.157
33	5.407	5.481	-0.074	4.924	0.483
34	5.353	5.152	0.201	5.503	-0.15
35	5.342	5.81	-0.468	5.596	-0.254
36	5.339	5.305	0.034	5.116	0.223
37	5.291	5.769	-0.478	5.46	-0.169
38	4.883	5.204	-0.321	4.813	0.07
39	4.726	5.342	-0.616	5.285	-0.559
40	4.591	5.324	-0.733	4.628	-0.037

Molecular docking details

Molecular docking was performed with Glide to explore the potential binding mode between the compunds and LSD1. Each compound was set to generate up to 32 conformations, and each conformation eventually generated up to 10 docking results in molecular docking. After that, the results with the higher docking scores and better superposition with ligand in the substrate region of the crystal structure (5LHI) were selected as the initial conformations for molecular dynamics simulation.

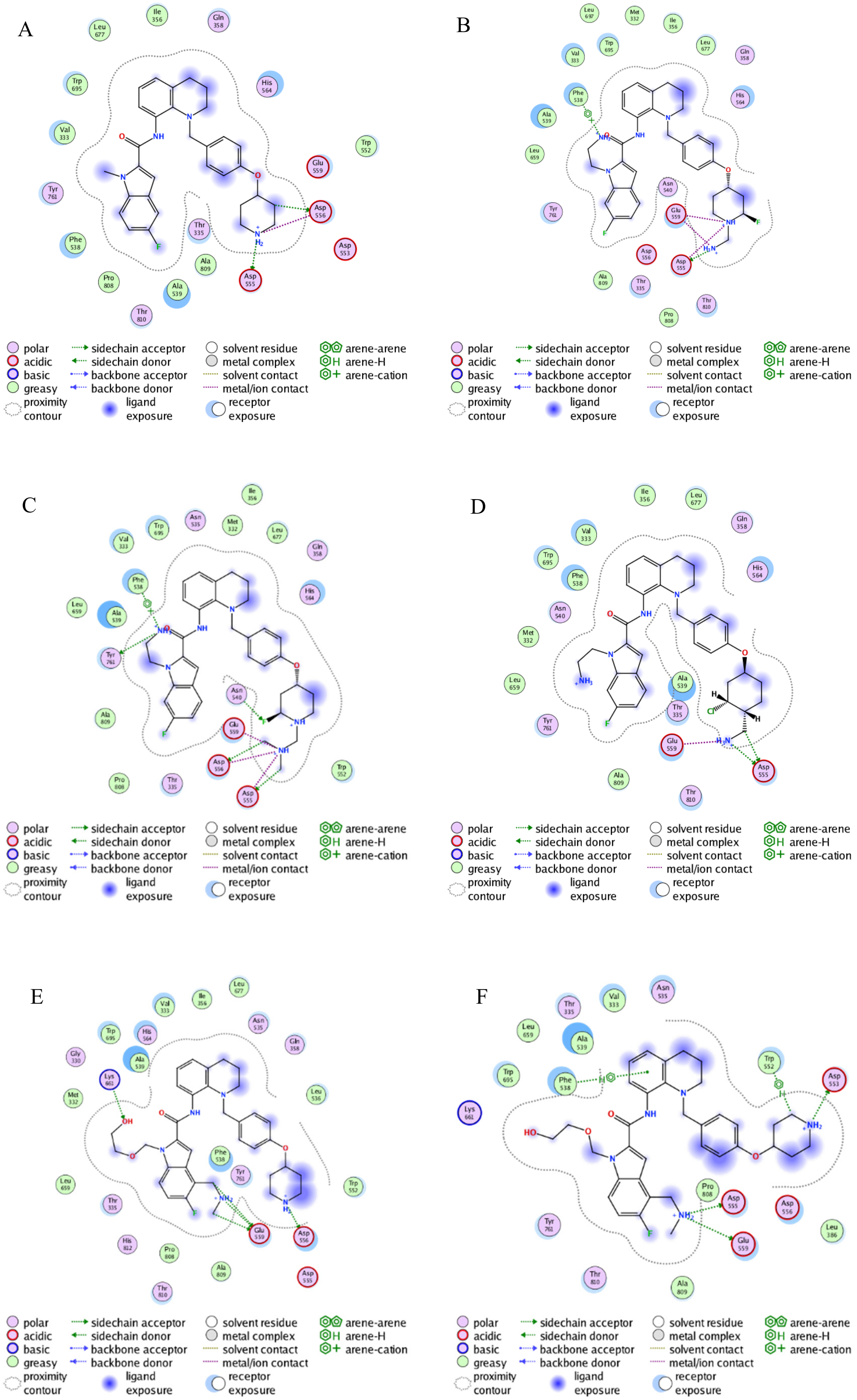

A
B
C
D
E
F
polar
acidic
basic
greasy
proximity contour
sidechain acceptor
sidechain donor
backbone acceptor
backbone donor
ligand exposure
solvent residue
metal complex
solvent contact
metal/ion contact
receptor exposure
arene-arene
arene-H
arene-cation

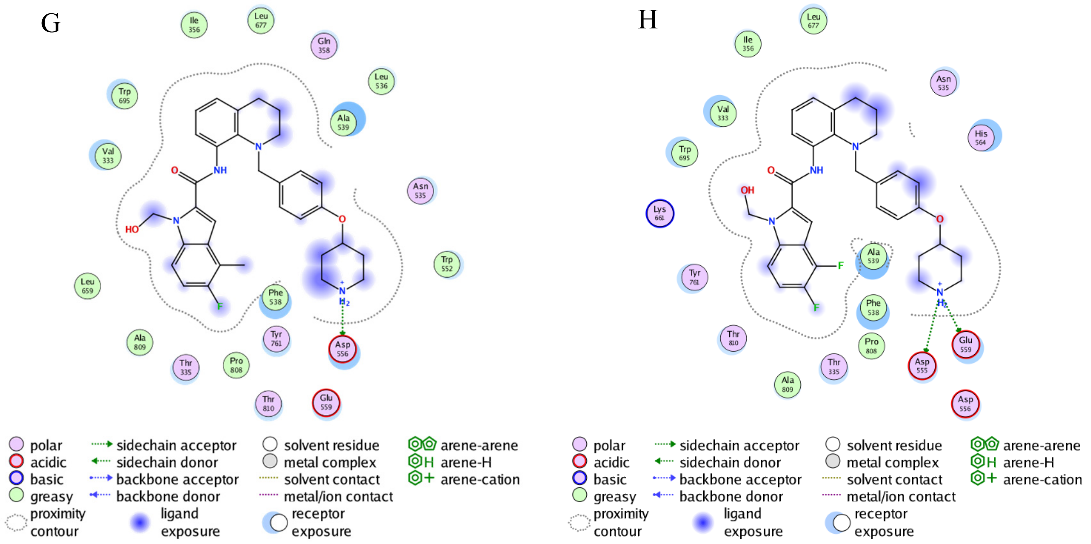

Figure S2. 2D diagrams of the interactions between the compounds and LSD1 (PDB code 5LHI). (A) compound 18x, (B) compound D1, (C) compound D2, (D) compound D4, (E) compound P8, (F) compound P56, (G) compound Z5, (H) compound Z17.

As shown in Figure S2, it was clear that the four compounds were all docked to the substrate binding site. These compounds were found to occupy a significant hydrophobic pocket consisting of residues Val333, Ile356, Phe538, Ala539, Leu677, Trp695 and Pro808 and keep a “U-shaped” conformation, which enhanced the binding stability of the compounds with LSD1. Particularly, the tetrahydroquinoline skeleton of each compound was surrounded by Val333, Ile356 as well as Trp695 and formed some hydrophobic interactions with these residues. In addition, there were strong polar interactions between these compounds and residues Thr335, Asp555, Glu559, His564, Tyr761 and Thr810. Separately, the protonated amine on the caudal six-membered ring of compound 18x could form a hydrogen bond with Asp555 and a salt bridge with Asp556 in figure S2A. The salt bridge was still present between compound D1 and Asp555 (Figure S2B), and a new salt bridge was found between compound D1 and Glu559. Particularly, the -NH_2 group attached to this six-membered ring formed a hydrogen bond with Asp555 and a salt bridge with Glu559, as with the -NH_2 group of compound D4 (Figure S2D). Differently, there was a unique arene-cationic interaction between the introduced amine on the indole ring of compound D1 and Phe538, not the compound D4. As can be seen from Figure S2H, the protonated amine on the compound Z17 formed a hydrogen bond with Asp555 in addition to a hydrogen bond with Glu559 rather than a salt bridge.

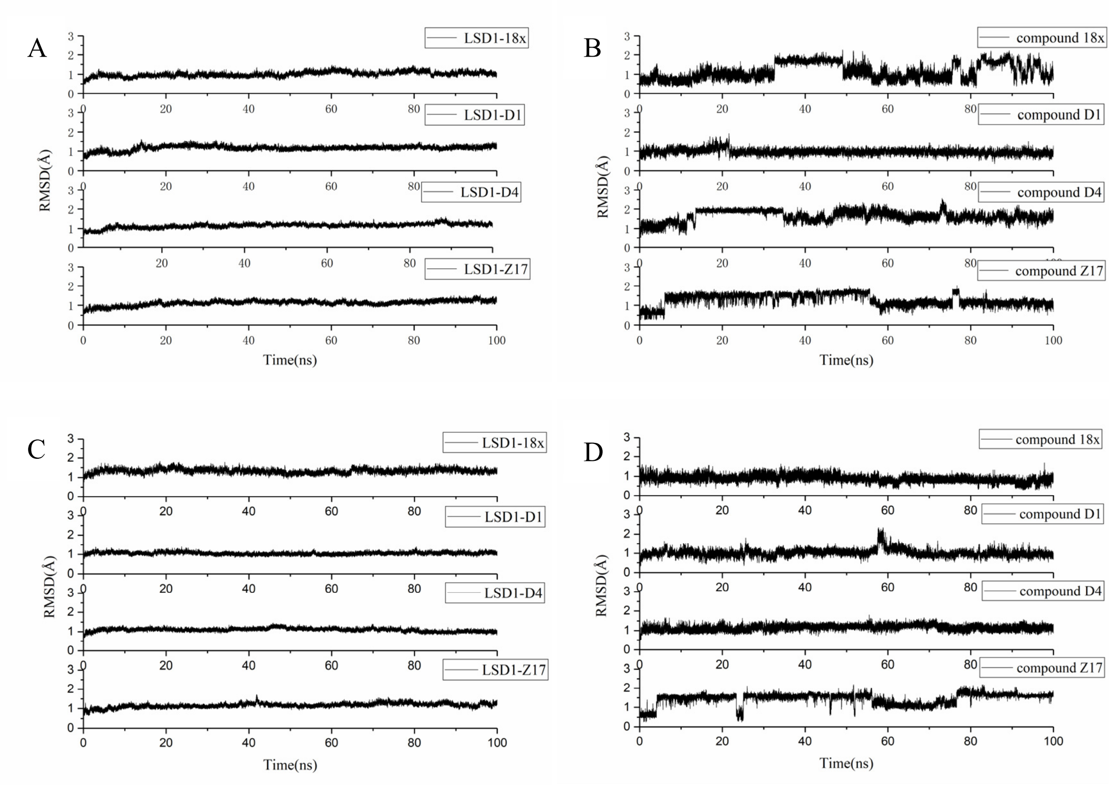

Figure 3S. The RMSD results for the second simulation and the third simulation. (A) and (B) are the RMSD values of the second simulated complexes and ligands, respectively. (C) and (D) represent the RMSD values of the third simulated complexes and ligands, respectively.

Table S2. Binding free energies of protein–ligand complexes from the first MD simulations. All the energies are in kcal/mol.

Contribution	LSD1-D1	LSD1-D4	LSD1-Z17	LSD1-18x
E_{vdW}	-52.06±3.62	-53.33±3.26	-58.97±3.15	-50.40±2.80
E_{ele}	-451.69±23.59	-274.64±13.12	-163.81±11.31	-164.74±13.91
G_{pol}	450.70±22.29	285.80±11.81	196.58±13.42	189.07±14.54
G_{np}	-5.38±0.12	-5.63±0.11	-5.36±0.13	-5.04±0.13
G_{bind}	-58.44±4.48	-47.80±4.42	-31.56±5.45	-31.11±3.74

Table S3. Binding free energies of protein–ligand complexes from the second MD simulations. All the energies are in kcal/mol.

Contribution	LSD1-D1	LSD1-D4	LSD1-Z17	LSD1-18x
E_{vdW}	-40.65±3.92	-56.21±3.24	-54.90±3.40	-46.89±2.81
E_{ele}	-510.20±22.97	-305.89±15.32	-165.98±13.59	-164.97±21.84
G_{pol}	505.19±21.95	324.05±13.45	199.67±14.48	190.28±20.79
G_{np}	-5.39±0.19	-5.77±0.11	-5.47±0.16	-5.18±0.17
G_{bind}	-51.05±5.54	-43.82±4.89	-26.68±4.48	-26.76±4.51

Table S4. Binding free energies of protein–ligand complexes from the third MD simulations. All the energies are in kcal/mol.

**Contribution**	**LSD1-D1**	**LSD1-D4**	**LSD1-Z17**	**LSD1-18x**
E_{vdW}	-39.85±4.30	-58.73±3.52	-55.62±3.37	-42.00±3.68
E_{ele}	-540.81±25.51	-261.59±13.13	-162.23±21.49	-153.38±13.49
G_{pol}	530.067±24.66	285.78±11.61	191.05±20.23	169.75±14.51
G_{np}	-5.78±0.23	-5.62±0.09	-5.22±0.16	-4.86±0.21
G_{bind}	-56.37±5.30	-40.16±4.84	-32.01±4.33	-30.48±3.69
